# Heart rate variability response of intensity-matched strength training dependent on body position in females: a pilot randomized crossover study: Autonomic responses of strength training depending on body position

**DOI:** 10.1038/s41598-025-19817-7

**Published:** 2025-09-15

**Authors:** Johannes Lässing, Florian Wegener, Nils Höpker, Kuno Hottenrott, Thomas Gronwald, Roberto Falz

**Affiliations:** 1 Department of Exercise Science & Sports Medicine, Martin Luther University Halle-Wittenberg, von-Seckendorff-Platz 2, 06120 Halle (Saale), Germany; 2 Institute of Interdisciplinary Exercise Science and Sports Medicine, MSH Medical School Hamburg, Am Kaiserkai 1, 20457 Hamburg, Germany; 3 Human‒Machine-Interaction, Magdeburg-Stendal University of Applied Science, Breitscheidstraße 2, 39114 Magdeburg, Germany; 4 Institute of Sports Medicine & Prevention, University Leipzig, Rosa- Luxemburg-Straße 30, 04109 Leipzig, Germany

**Keywords:** Autonomic control, Posture, Orthostasis, Intensity-matched exercise, Vagal withdrawal, Resistance training, Cardiology, Health care, Medical research, Physiology

## Abstract

Heart rate variability (HRV), as an indicator of autonomic control, has been rarely studied during strength training application. This study investigates the acute HRV responses to intensity-matched resistance exercises, targeting similar muscle groups but performed in different body positions. Fourteen healthy females (21.6 ± 2.0 years) performed a 3-repetition maximum test (3-RM) for the squat movement in the Smith machine (SM, upright) and the leg press (LP, seated). During two subsequent visits, they randomly completed two exercise sessions in SM and LP (two sets of 10 repetitions at 50% 3-RM). HRV was assessed continuously (via ECG) throughout the interventions.

At pre-exercise, the Root Mean Square of Successive Differences (RMSSD) and RR intervals (RRI) were significantly higher for the LP condition. Alpha1 of Detrended Fluctuation Analysis (DFAa1) was significantly higher with SM at rest. During exercise sessions, the LP condition revealed significantly reduced RMSSD (∆exercise: SM -1.19 ± 14.57 ms vs. LP -22.23 ± 22.46 ms; *p* = .013) and higher RRI (*p* < .001). No differences were observed for DFAa1. The changes between pre- and post-exercise for RMSSD and DFAa1 showed no differences between LP and SM conditions; however, RRI was significantly reduced for SM (*p* = .008). Within the conditions, RMSSD decreased significantly from pre- to post with SM (*p* 0.008) but not with LP (*p* = 0.271). Squats within the SM condition led to increased vagal withdrawal both at rest and after exercise, probably due to orthostatic stress. While exercising, the differences in autonomic regulation are less noticeable. Future analyses should examine the body position-dependent HRV responses to post-exercise hypotension.

## Introduction

Strength training exercises are established for both the prevention and therapy of cardiovascular disease^1,2^with lower extremity strength training exercises performed in a seated position, preferred to minimize cardiovascular risks^3,4^. The acute regulation of blood pressure, mediated by autonomic nervous system activity via efferent sympathetic and parasympathetic neural pathways, is crucial for maintaining cardiovascular stability during changing external conditions such as mechanical loads^5,6^. However, research shows that strength training can cause a temporary decrease in blood pressure after exercise, known as post-exercise hypotension^7–11^. It appears that the intensity of exercise is crucial for the degree of post-exercise blood pressure reduction. Moreover, the response of blood pressure during strength training is influenced by body position, making it a significant factor in assessing risks associated with strength training in cardiac rehabilitation^3,4^. Farinatti et al. (2021) discovered that post-exercise hypotension is linked to an increase in the sympathetic nervous system activity and a decrease in the parasympathetic nervous system activity^8^. This raises the question of the influence of body position on autonomic regulation during and after strength training.

Unlike autonomic regulation under resting conditions in response to orthostasis^12–14^the mechanisms governing autonomic regulation in various body positions during strength training and different exercise selections are often debated^7,15–19^. The differences in the implementation protocols and the timing of the measurements seem to be the primary reasons for the discrepancies observed. Inter-beat interval (RR) measurements for heart rate variability (HRV) analysis are mainly based on pre- and post-comparisons tests in resting periods, which are conducted in either a sitting^7,15,16^ or lying position^17,20,21^. However, a withdrawal of parasympathetic activity and an increase in sympathetic activation after strength training, which mainly depends on the intensity, is generally accepted as a basic observation^8,18,19^.

Only a limited number of studies have thoroughly examined HRV metrics during strength training exercises, highlighting a significant gap in our understanding of this vital physiological response^6,21^. Weippert et al. (2013) found that a static strength exercise with high cardiovascular demands, such as the leg press, generated greater cardiac parasympathetic activity during exercise than a dynamic leg exercise using a cycle ergometer^21^. Iglesias-Soler et al. (2015) suggest that standing squat exercises combined with more intense cardiovascular regimes tended to result in higher vagal withdrawal during exercise than those with lower cardiovascular demands^6^. The influence of differences in body position on these results has not yet been addressed and is the focus of the present study.

To our knowledge, there is no existing research that outlines the differences in acute autonomic regulation based on body position during strength training. Enhancing our understanding of how body position affects acute autonomic regulation during muscular demands could improve the safety of strength training for individuals with autonomic dysfunction or cardiovascular disease. Therefore, this study aims to investigate the effects of strength training on autonomic regulation in relation to body position, specifically during a standing exercise using the Smith machine (SM) and a seated inclined exercise using the leg press (LP). We assume that standing before and during exercise results in a significantly greater level of parasympathetic inhibition. Furthermore, performing strength training in a standing position will enhance the inhibition of vagally mediated HRV parameters after the training session is completed.

## Methods

### Participants

The study was conducted (between November 2023 and May 2024) in accordance with the latest version of the Declaration of Helsinki and was approved by the Ethics Committee of the Medical Faculty at the University of Halle (Saale) (approval number: 2023–202). The participants were female players from a local handball sports club with a weekly training volume of 9.0 ± 1.0 h, including approximately 1.5 h of circuit strength training. All participants provided written informed consent to participate in the study before the examinations. For study inclusion, participants had to regularly attend the club’s training sessions and matches and be free of cardiac, pulmonary, or inflammatory diseases. ECG monitoring was employed for ensuring cardiac safety during investigations. No adverse events were reported for any of the participants in the study. Participants were also excluded if orthopaedic anomalies were identified during the baseline assessments. A total of 14 healthy, trained female players were recruited (age, 21.6 ± 2.0 years; body mass, 68.9 ± 6.5 kg; body height, 170.4 ± 4.8 cm; body mass index, 23.6 ± 1.6 kg/m^2^; body fat mass, 27.9 ± 4.2%; body muscle mass, 47.0 ± 3.2%). Due to a lack of sufficient literature on this specific question, it was not possible to calculate the number of cases. Given the exploratory nature of our study, the sample size was determined based on the recommended minimum requirement of 12 subjects for pilot studies^22^. Data on the SDNN response to postural changes without exercise indicated that a sample size of at least 10 participants was needed (SDNN values: sitting 45.4 ± 6 vs. standing 38.2 ± 4; effect size d = 0.82; power = 0.8; alpha = 0.05)^12^.

## Study design

This study is part of a larger investigation focused on the effects of orthostasis-dependent hemodynamic regulation in the cardiovascular system. The details of the test setup and measurement methods have already been thoroughly described. This presentation will expand upon those details by including heart rate variability (HRV) measurements for the second part of the study^4^.

**Fig. 1:**
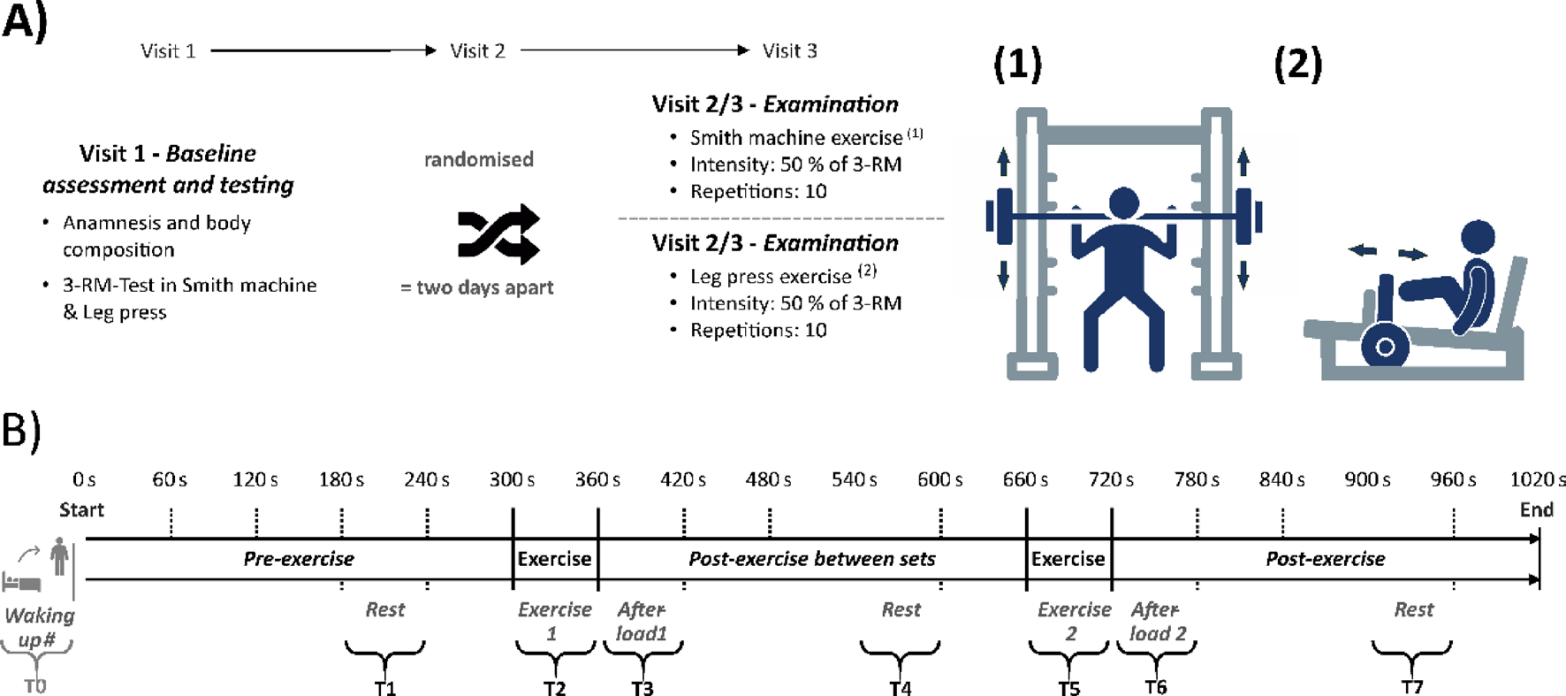
(**A**) Timeline of study period; (**B**) Timeline of the 2nd and 3rd visit with squats in the Smith machine (1) and leg bends in the leg press (2). Abbreviations: RM (repetition maximum); s (seconds); T0 (baseline measurement); T1/T4/T7 (rest measurements 1 min); T2/T5 (exercise measurements 1/2); T3/T6 (afterload measurements 1/2); Pre-exercise /Post-exercise between sets /Post-exercise measurements 5 min.

Briefly, this randomized crossover study involves a baseline assessment (visit 1) followed by two training sessions conducted in two different positions (visit 2 and 3): standing (SM) and seated at a 60° incline (LP) as shown in Fig. 1. Participants were randomly assigned to groups: Group A started with LP, while Group B started with SM. This randomization utilized a computer-generated block sequence (https://miniwebtool.com/de/listen-randomisierer/) with a fixed block size of four. To maintain the confidentiality of the allocation, an independent colleague conducted the randomization process. Due to the nature of the intervention, it was not feasible to blind participants or researchers providing exercise instructions regarding group allocation. In addition to the previously described protocol, participants underwent a 3-minute RR interval recording and HRV analysis (RS800, Polar^®^, exported via Polar Flow desktop software) in a lying position immediately upon waking on both training days (Fig. 1, B-T0). The baseline assessment also included medical history, lifestyle questionnaire, physical activity status, tobacco and alcohol use, body composition (bioelectrical impedance analysis, Tanita BC-545 N), and determination of the 3-repetition maximum (3-RM) for SM (Technogym) and LP (Selection 700, Technogym) using the approximation method. The 3-RM tests were conducted following the guidelines from the American College of Sports Medicine (ACSM) for 1-RM testing^23^; briefly, participants completed incremental tests in both the SM and LP, ensuring a standardized knee flexion of 90°. During block randomized visits 2 and 3, scheduled at the same time of day with at least two days of recovery, participants completed strength training sessions using either the SM or LP. Each strength exercise session consisted of two sets of 10 repetitions at 50% 3-RM, performed at a controlled movement velocity (starting point: extended knee and hip joints; 2s eccentric, 0s pause, 2s concentric, 2s pause – 2-0-2-2) and breath control (inhaling during the eccentric phase and exhaling during the concentric phase), with visual and auditory feedback provided (Interval Timer, dreamspark). The RR intervals were continuously recorded during visits 2 and 3. Before each visit, participants were instructed to avoid any lower-body strength training for 48 h and were advised to avoid caffein and alcohol intake during this period. Out of fourteen players, nine used hormonal contraceptives while five did not. No phases of the menstrual cycle were surveyed. During the study visits, none of the participants reported any physical complaints.

Taking into account the influence of the circadian rhythm on physiological metrics such as HRV, the participants were always recruited at the same time of test day (temporal mean deviations of the respective measurement starts; 0:42 ± 0:03 h: min). The measurements for visits 2 and 3 were on average 3.3 (± 1.9) days apart.

## RR interval recording and HRV analysis

For the LP and SM conditions, the resting position was chosen as upright standing for SM and seated with an inclined angle of 90° at the knee joint in LP. RR intervals were recorded continuously using an ECG Task Force monitor (Task Force Monitor 340i, CNSystems Medizintechnik GmbH, Austria). The study follows the standard guidelines established by the Task Force of the European Society of Cardiology in 1996^24^. For resting measurements breathing was chosen spontaneously to avoid the influence of paced breathing. This registered medical device with a sampling rate of 1000 Hz is established and validated for assessing RR intervals and other metrics and has been used in previous studies (Task Force of the European Society of Cardiology and the North American Society of Electrophysiology^25^. For preparation, the skin was exfoliated and disinfected, and electrodes for a 6-lead chest ECG were applied according to standard procedures. Before each strength training session, the device was calibrated according to the manufacturer’s instructions. HRV data were analysed using Kubios HRV analysis software (Kubios HRV Scientific Lite 4.1.0, Biosignal Analysis and Medical Imaging Group, Department of Physics, University of Kuopio, Kuopio, Finland). Pre-processing settings were set to the default values, including the RR detrending method, which was kept at “smoothness priors” (Lambda = 500). The RR interval series was then corrected using the Kubios HRV “automatic correction” method^26^. RR interval length (RRI in ms), the root mean square of successive differences (RMSSD in ms), the ratio of the low-frequency (LF in ms^2^) and high-frequency (HF in ms^2^) band (LF/HF), and the short-term scaling exponent alpha1 of Detrended Fluctuation Analysis (DFAa1) were derived. Frequency-domain LF/HF ratio was analyzed via Fast Fourier-Transformation (FFT) only for 5-min recordings during resting conditions pre- and post-exercise. The LF band was set at 0.04 to 0.15 Hz, and the HF band at 0.15–0.40 Hz. DFAa1 as a non-linear metric was calculated as the root mean square (RMS) fluctuations of the integrated and detrended RR-intervals analyzed in observation windows of different sizes and then further processed as the slope between the RMS-fluctuation data in relation to the different window sizes on a log-log scale (Peng et al., 1995). The Window size was set to 4 ≤ *n* ≤ 16 beats in the software preferences. Data were also visually scanned for artefacts (e.g., spikes in RR-interval series which were not corrected by the artifact correction algorithm), marked as “noise” and removed manually. Data sets with > 5% artifacts were excluded from HRV analysis.

For analysis and comparison of RRI, two fixed intervals were selected: 1-minute intervals (Fig. 1, B: T1 – T7) to examine dynamic autonomic responses during exercise and rest, and 5-minute intervals (Fig. 1, B: Pre-exercise – Post-exercise) to assess pre-post exercise resting states^6,12,15^.

To represent dynamic changes, we averaged the 1-minute mean values of the exercise (T2, T5), afterload periods (T3, T6), rest periods before (T1, T4), and post-exercise (T4, T7). Delta values represent differences between the consecutive periods (∆values = mean values of the current period – mean values previous period). Additionally, differences between pre- and post-exercise periods were analysed. To assess immediate intra-individual responses after exercise, mean differences between the first and last rest periods were compared for each strength training session.

### Statistical analysis

Unless otherwise stated, all values are expressed as means ± standard deviations. Statistical significance was set at *p* < .05, and all tests were two-tailed. Descriptive data processing was performed using Microsoft Excel^®^ 2021 for Windows (Microsoft Corporation, Redmond, Washington, USA), while all statistical analyses were conducted in GraphPad Prism 9 (GraphPad Software Inc., California, USA). Normal distribution was assessed using the Kolmogorov-Smirnov test. For normally distributed data, comparisons between sessions were performed using two-way repeated-measures ANOVA with Bonferroni post hoc correction for multiple comparisons. HRV changes (∆values = mean values current period – mean values previous period) were analysed using two-way repeated-measures ANOVA. Within-group differences for baseline tests, pre-post comparisons of the five-minute periods, and intra-individual group changes were assessed using paired Student’s t-tests. Non-normally distributed data were analysed with the non-parametric Wilcoxon matched-pairs signed rank test.

## Results

### Baseline parameters

Mean baseline parameters recorded immediately upon awakening on the designated test day are presented in Table 1. Due to recording artefacts, two participants were excluded from the three-minute analysis and one from the one-minute analysis. The results show that there are no differences in the baseline parameters between the LP and SM conditions.

**Table 1:** Mean baseline parameters of HRV upon awakening in SM and LP conditions.

Baseline HRV	SM	LP	*p* value	η^2^_*p*_
HR in bpm (n = 12)	63.4 ± 4.4	61.2 ± 7.4	*p* = .563	
RRI in ms (n = 12)	950.7 ± 71.1.10	980.0 ± 111.3	*p* = .339	
RMSSD in ms (n = 12)	105 ± 36	107 ± 39	*p* = .818	0.005
DFAa1 (n = 13)	0.90 ± 0.32	0.89 ± 0.24	*p* = .968	< 0.001

The values are presented as the means and standard deviations, paired Student’s t-test η²_p_ = partial eta square or Wilcoxon matched-pairs rank test; 3-RM = three-repetition maximum test; SM = Smith machine; LP = leg press, HR = heart rate, RRI = RR interval, RMSSD = Root-Mean-Square-of-Successive-Differences, DFAa1 = short-term scaling exponent alpha1 of Dentrended Fluctuation Analysis.

## HRV metrics during exercise, and pre-post-exercise periods in SM and LP (1-minute periods)

**Fig. 2:**
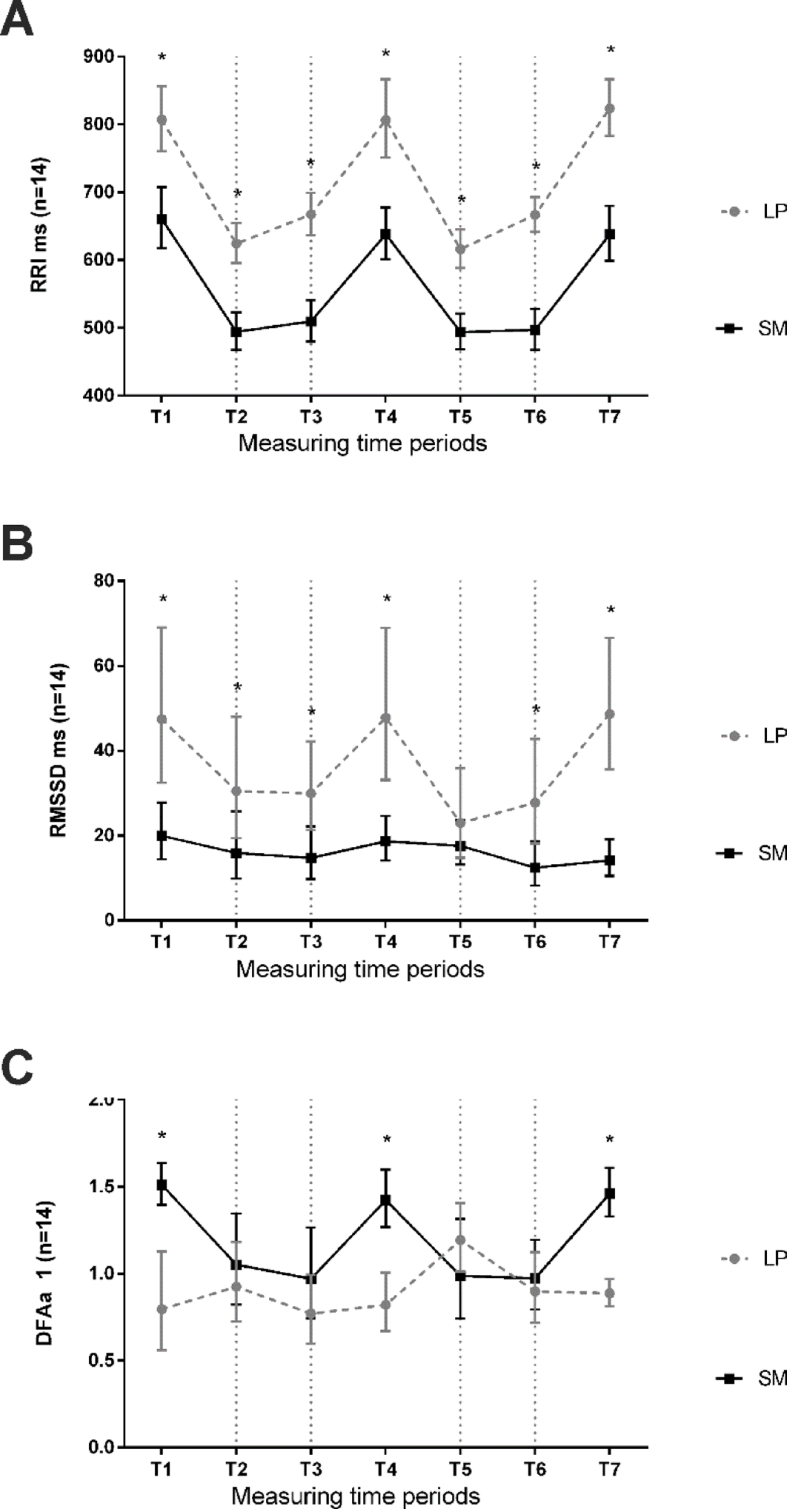
shows the 1-minute evaluations of the HRV metrics for the rest periods (T1, T4, T7), for the exercise periods (T2, T5), and for the immediate afterload periods (T3, T6).

Figure 2. *RRI (A)*,* RMSSD (B)*,* and DFAa1 (C) in comparison of both conditions*,* SM and LP*,* throughout the 1-minute time periods (n = 14) T1 = Rest 1*,* T2 = Exercise 1*,* T3 = Afterload 1*,* T4 = Rest 2*,* T5 = Exercise 2*,* T6 = Afterload 2 and T7 = Rest 3)*,* RRI = RR intervals*,* RMSSD = Root-Mean-Square-of-Successive-Differences*, DFAa1 = short-term scaling exponent alpha1 of Detrended Fluctuation Analysis. * post hoc analysis with significant differences p < .05.

Figure 2 presents that for RRI, there were significantly higher values for the LP condition compared to SM for each period (group: F = 168.70, *p* < .001; time: F = 96.01, *p* < .001; interaction: F = 3.52, *p* = .004; η²_p_ = .213; all post-hoc tests *p* < .001). For DFAa1, significantly higher values were observed during the rest periods (T1, T4, and T7) in SM condition compared to LP condition (group: F = 18.26, *p* < .001; time: F = 3.99, *p* < .001; interaction: F = 8.89, *p* < .001; η²_p_ = .406; all post-hoc tests *p* < .001). RMSSD showed significantly higher values for the LP condition compared to SM for all periods except for the second exercise period (T5) (group: F = 15.64, *p* = .002; time: F = 3.46, *p* = .004; interaction: F = 6.20, *p* < .001; η²_p_ = .323; T1, T4, T6 and T7 post-hoc tests *p* < .001, T2: *p* = .001, T3: *p* = .006, T5: *p* = .261).

Table 2 shows the changes in the respective parameters for conditions LP and SM as a function of the previous period.

**Table 2:** Mean change in 1-minute HRV metrics compared with the previous measurement time point (∆values = mean values current period – mean values previous period; *n* = 14).

	SM 50% 3-RM	LP 50% 3-RM		Effect size for interaction effects	Main effects (group, time, interaction)		
∆ HRV metrics			*p-value/post hoc-test*	*η²*_*p*_	*group* *F value; p value*	*time* *F value; p value*	*interaction* *F value; p value*
RRI in ms ∆ Exercise ∆ Afterload ∆ Rest	-157.1 ± 60.20 9.67 ± 34.95 135.6 ± 35.11	-189.5 ± 85.71 46.32 ± 30.67 151.0 ± 66.90	0.322 0.210 > 0.999	.*203*	**F = 9.56;** *p* = .009	**F = 108.8;** *p* < .001	F = 3.32; *p* = .052
RMSSD in ms ∆ Exercise ∆ Afterload ∆ Rest	**-1.19 ± 14.57** -3.37 ± 14.08 **1.12 ± 8.92**	**-22.23 ± 22.46** 0.52 ± 21.47 **21.16 ± 18.96**	**0.013** > 0.999 **0.018**	*0.424*	F = 1.29; *p* = .276	**F = 6.46;** *p* = .005	**F = 9.56;** *p* < .001
DFAa1 ∆ Exercise ∆ Afterload ∆ Rest	**-0.38 ± 0.34** -0.06 ± 0.23 **0.42 ± 0.31**	**0.23 ± 0.31** -0.22 ± 0.29 **-0.01 ± 0.20**	**< 0.001** 0.769 **0.013**	*0.541*	F = 0.08; *p* = .783	**F = 8.16;** *p* = .001	**F = 15.34;** *p* < .001

The values are presented as the means and standard deviations, F = Effect size according to Cohen’s two-way repeated-measures ANOVA; η²_p_ = partial eta square for interaction effect; ∆ Exercise (mean value Exercise T2/T5 – mean value Rest T1/T4), ∆ Afterload (mean value Afterload T3/T6 – mean value Exercise T2/T5), ∆ Rest (mean value Rest T4/T7 – mean value Afterload T3/T6); RRI = RR intervals, RMSSD = Root-Mean-Square-of-Successive-Differences, DFAa1: short-term scaling exponent alpha1 of Detrended Fluctuation Analysis.

Significant values are in bold.

## HRV metrics at pre-post-exercise periods in SM and LP (5-minute periods)

One subject was excluded from the analysis due to an artefact rate > 5%. Table 3 compares LP and SM for the respective rest periods over 5-minute periods.

**Table 3:** Mean values of 5-minute resting periods pre- and post-exercise (two-way ANOVA with post hoc test; *n* = 13).

	SM 50% 3-RM	LP 50% 3-RM		Effect size for interaction effects	Main effects (group, time, interaction)		
HRV metrics			*p-value/post hoc-test*	*η²*_*p*_	*group* *F value; p value*	*time* *F value; p value*	*interaction* *F value; p value*
RRI in ms Pre-exercise Post-ex. b/w sets Post-exercise	**662.70 ± 69.02** **620.80 ± 69.21** **604.50 ± 63.73**	**791.00 ± 72.03** **771.90 ± 55.87** **775.90 ± 50.05**	**< 0.001** **< 0.001** **< 0.001**	*0.382*	**F = 82.61;** *p* < .001	**F = 16.43;** *p* < .001	**F = 8.52;** *p* = .003
RMSSD in ms Pre-exercise Post-ex. b/w sets Post-exercise	**22.30 ± 9.82** **23.80 ± 10.19** **17.32 ± 6.08**	**68.09 ± 39.30** **60.06 ± 33.58** **57.40 ± 32.75**	**< 0.001** **< 0.001** **< 0.001**	*0.060*	**F = 21.25;** *p* < .001	F = 2.02; *p* = .155	F = 0.76; *p* = .478
DFAa1 Pre-exercise Post-ex. b/w sets Post-exercise	**1.42 ± 0.18** **1.30 ± 0.20** **1.40 ± 0.27**	**0.87 ± 0.25** **0.88 ± 0.21** **0.90 ± 0.19**	**< 0.001** **< 0.001** **< 0.001**	*0.139*	**F = 78.67;** *p* < .001	F = 1.41; *p* = .263	F = 1.94; *p* = .166
LF/HF Pre-exercise Post-ex. b/w sets Post-exercise	**5.65 ± 4.78** **3.11 ± 1.89** **3.35 ± 2.57**	**0.97 ± 0.60** **0.94 ± 0.62** **0.90 ± 0.62**	**< 0.001** **0.024** **0.001**	*0.222*	**F = 20.03;** *p* < .001	**F = 3.44;** *p* = .049	**F = 3.43;** *p* = .049

The values are presented as the means and standard deviations, F = Effect size according to Cohen’s two-way repeated-measures ANOVA; η²_p_ = partial eta square for interaction effect; Post-ex. b/w sets = post-exercise between sets, RRI = RR intervals, RMSSD = Root-Mean-Square-of-Successive-Differences, DFAa1: short-term scaling exponent alpha1 of Detrended Fluctuation Analysis, LF/HF = ratio of low frequency and high frequencies.

Significant values are in bold.

Table 4 shows the differences between the first (Pre-exercise) and the last rest period (Post-exercise) over five minutes for the LP and SM conditions.

**Table 4:** Comparisons of HRV metrics for 5-minute pre- and post-exercise (∆values = mean values post-exercise – mean pre-exercise; *n* = 13).

	SM 50% 3-RM	LP 50% 3-RM		Effect size
∆ HRV metrics			*p value*	η²_p_
RRI in ms ∆ pre-post	**-58.22 ± 5.30**	**-15.12 ± 21.98**	**0.008**	0.458
RMSSD in ms ∆ pre-post	-4.98 ± 5.61	-10.68 ± 33.37	0.893	-
DFAa1 ∆ pre-post	-0.02 ± 0.09	-0.03 ± 0.05	0.679	0.015
LF/HF ∆ pre-post	**-2.31 ± 3.59**	**-0.06 ± 0.61**	**0.049**	0.290

The values are presented as the means and standard deviations, paired Student’s t-test, η^2^_p_ = partial eta square, or Wilcoxon matched-pairs rank test, ∆ pre-post = mean value last post-exercise period (5-min.) – mean value pre-exercise (5-min); RRI = RR intervals, RMSSD = Root-Mean-Square-of-Successive-Differences, DFAa1: short-term scaling exponent alpha1 of Detrended Fluctuation Analysis, LF/HF = ratio of low frequency and high frequencies.

Significant values are in bold.

### Intra-individual pre-post differences per group (5-minute periods)

**Fig. 3:**
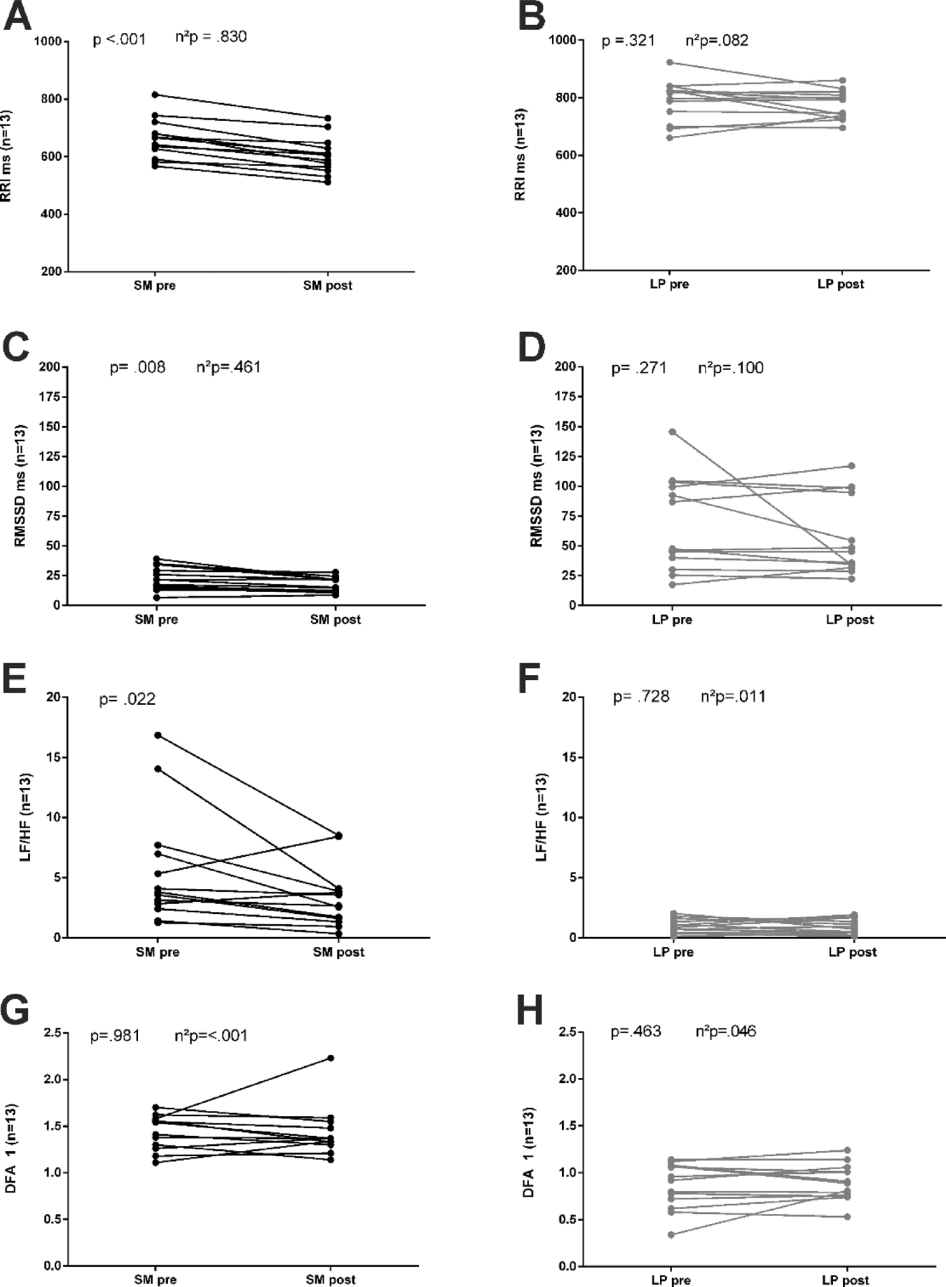
Differences between the 5-minute periods pre- and post-exercise for each condition; (**A**) and (**B**) (RRI = RR intervals), (**C**) and (**D**) (RMSSD = Root-Mean-Square-of-Successive-Differences), E and F (LF/HF = ratio of low frequency) and G and H (high frequencies DFAa1: short-term scaling exponent alpha1 of Detrended Fluctuation Analysis).

The intra-individual differences between the pre- and post-exercise periods are shown in Fig. 3.

In LP (Panels B, D, F, H), no significant pre-post differences were observed in any of the parameters. In LP (Panels B, D, F, H), no significant pre-post differences were observed in any of the parameters. For SM, significant pre-post differences were observed for the parameters RRI, RMSSD, and LF/HF (3 A, 3 C and 3 E).

## Discussion

The main results of the present study indicate that a seated position in the LP condition leads to significantly higher vagally-mediated HRV (higher RMSSD values) and higher RRI during the resting phase. Vagal withdrawal significantly increased during leg strength exercises under the LP condition; however, the RRI did not reach the lower levels observed in the standing SM condition. In the subsequent, directly afterload phase, significantly higher vagally-mediated RMSSD values are observed for the LP condition, which returns to the baseline level during the extended post-exercise phase. A pre-post comparison of RMSSD (Table 4) reveals no difference between the two conditions, indicating that body position has a significant influence on autonomic regulation in both the pre-exercise and post-exercise states (Table 3). However, in the within-subject comparison (Fig. 3), the SM condition shows a significantly greater reduction in RMSSD, which can be interpreted as strength training-induced parasympathetic withdrawal in the standing post-exercise phase.

### Baseline and pre-exercise regulation

Baseline HRV assessment in the lying position immediately after awakening revealed no differences in the respective HRV parameters. Directly before the start of training sessions, significant differences were observed. The current literature consistently shows that a standing position, compared to a sitting position, leads to higher heart rate and thus to a shorter RRI in healthy individuals^12–14^.

Souza et al. (2014) found that changing from a sitting to a standing position decreased parasympathetic activity while increasing sympathetic system influence on heart rate^12^. Consistent with the current literature, significantly lower RRI and RMSSD values were observed in the pre-exercise period with SM. The response of the autonomic nervous system (ANS) in relation to a standing position is based on an initial withdrawal of parasympathetic influence and activation of the sympathetic branch, resulting in an increased heart rate^12–14^. De Souza et al. (2014) indicate that the short-term scaling exponent DFAa1 is highly sensitive for assessing autonomic position-dependent changes in young healthy females, with significantly greater expression observed when standing compared to sitting^12^. Consistent with de Souza et al. (2014), our study showed that DFAa1 is significantly higher for SM compared to LP, indicating higher correlation properties in heart rate time series with less complexity^27^.

Additionally, a notably higher LF/HF ratio for SM was observed under resting conditions (before training), which primarily indicates an altered sympathetic-parasympathetic balance with increased parasympathetic withdrawal^13^. Thus, the available data suggest a possible baroreflex-induced increase in heart rate and higher organismic demands for SM ^12–14^.

### Regulation during strength exercise in SM and LP

The corresponding published study by Lässing et al. (2025) demonstrated significantly higher heart rates and blood pressure responses during exercise in SM ^4^. Following Katayama et al. (2018), the observed higher relative intensity (lower work efficiency rate) and the higher metabolic effort (VO_2_ and VCO_2_) in SM could reverse the baroreflex-induced inhibition of muscle sympathetic nerve activity. This may clarify the noticeably greater increase in heart rate during exercise within the SM condition^28^. However, as the shown ratio of heart rate increase to mean ground reaction force (∆heart rate/force output) did not differ between conditions, the authors hypothesized that body position had a significant influence on heart rate^4^. In line with the study by de Sousa et al. (2014), initial exercise analysis shows significantly higher RMSSD values during the first set (T2) in the LP condition. However, the mean values during the second set (T5) do not exhibit substantial differences between the conditions in the present study. Level of intensity, break time, and number of repetitions (e.g., time under tension) influence autonomic modulation^18^. Therefore, it seems plausible that parasympathetic withdrawal increases during the second performance in LP^9,10,29^.

Weippert et al. (2013) investigated the differences in autonomic regulation at matched RRI values during static squat performance (moderate weight) in a lying leg press and a dynamic moderate leg exercise using a cycle ergometer in a seated position. In accordance with our findings, significantly higher RMSSD parameters were found in the supine position. The authors proposed that static strength exercises (performed in a lying position) increase cardiac vagal activity compared to dynamic exercises (performed in a sitting position). However, the authors assumed a higher level of sympathetically induced peripheral vasoconstriction during supine leg press exercise. The authors attribute this to the higher blood pressure responses observed and the significantly higher pressure-frequency product in the leg press condition^21^. Lässing et al. (2025a) found no significant differences in total peripheral resistance (TPR) values between SM and LP during exercise. An increase in TPR during the performance of strength exercises, especially isometric strength stimuli, is well documented and is due to mechanical blood vessel compression, pressor reflex responses, and central feedforward mechanisms^10,30–33^. Weippert et al. (2013) reported lower cardiac complexity values and higher regularity (as indicated by approximate entropy and sample entropy) during static strength exercise, hypothesizing an increased metaboreflex response as the cause for the higher peripheral sympathetic activity. However, Laginestra et al. (2023) showed in a leg press exercise that the metaboreflex response forces an overall sympathetic activation, accompanied by parasympathetic cardiac withdrawal. Further studies confirm this view, showing that higher muscular strength demands are associated with a withdrawal of vagal influence or a resetting of the baroreflex^6,15,18,34,35^. Thus, the higher cardiac parasympathetic activity observed in the study by Weippert et al. (2013) is most likely explained by the different body positions adopted during the selected exercises.

Zhang et al. (2009) reported that blood pressure and RRI fluctuations during position changes in a single squat-stand manoeuvre are mediated by the baroreflex mechanism. In that regard with the present data, we can observe a more substantial decrease in DFAa1 values for the exercise period during SM conditions which can be interpreted as a change in correlation properties of heart rate time series^27^and can be caused by body position changes and thus higher variations in baroreflex regulation during standing SM^5^. For the LP condition, we observed no differences in correlation properties during exercise. Therefore, autonomic regulation in the LP is characterized by an increased withdrawal of parasympathetic activity, which does not reach the level of the SM condition^15,18,36^.

### Afterload-exercise regulation (1-min afterload)

The present data indicate significantly higher RRI and RMSSD values for LP during the first minute after the onset of afterload. Thus, the vagal influence is more pronounced when performing LP. However, there were no differences in the hemodynamic afterload regulation for the parameters ∆ afterload RRI, ∆ afterload RMSSD, and ∆ afterload DFAa1 between the conditions. This indicates that there are no differences in the autonomic regulation immediately after exercise, as shown in Table 2. In the immediate afterload regulation, the study by Lässing et al. (2025) showed that heart rate was significantly higher during the SM condition^4^. However, there were no differences in heart rate regulation (∆ afterload heart rate = 1 min afterload − 1 min exercise) during the first minute of the afterload period, too. The authors speculated that an insufficient chronotropic response to post-exercise recovery may prevent post-exercise hypotension^4^. In that regard, Farinatti et al. (2021) showed that post-exercise hypotension is associated with an increased vagal withdrawal and an increase in sympathetic activity. The authors conclude that autonomic regulation is not the main mechanism responsible for post-exercise hypotension^8^. In agreement with Farinatti et al. (2021), there is no evidence that the observed more pronounced reduction in blood pressure in SM during afterload can be primarily attributed to lower vasomotor nerve activity. Nevertheless, Lässing et al. (2025) showed an acute drop in TPR for SM in the immediate afterload phase, which was associated with higher relative intensities due to greater metabolic and stabilization demands^4^. Consequently, a decrease in TPR would result from an increased metabolic response in SM, which would mask the higher sympathetic influence on the vasomotor system shown above^37,38^.

There are also no differences in the immediate change of the fractal correlation properties of heart rate time series (∆ afterload DFAa1) in comparison of SM and LP. The authors of this study propose that the dominant vagal activity observed in the LP condition is not a consequence of autonomic load or direct afterload regulation but rather reflects the higher vagal activity present in the resting (pre-exercise) state.

In summary, the autonomic regulation in the first minute of the afterload period was not significantly different, which does not explain the corresponding observations of a reduction in TPR and, consequently, a blood pressure response for SM^4^. Nevertheless, LP demonstrates a greater parasympathetic influence, likely due to body position during the pre-exercise period.

### Post-exercise regulation (5-min pre-post comparison)

Lässing et al. (2025) demonstrated that body position during strength training—whether upright or seated in inclined position—affects heart rate and the regulation of blood pressure after exercise. The elevation in heart rate can be viewed as a compensatory mechanism to address the considerable reduction in vascular resistance that occurs upon standing^4^. Regardless of body position, most current literature describes that strength training in pre-post comparisons leads to parasympathetic withdrawal and increased sympathetic activation^8,18,19^. These studies show that autonomic regulation is predominantly dependent on the level of intensity. The reviews suggest that changes occur within 30 min after exercise, regardless of the training protocol or measurement parameters used. They collectively acknowledge that current data availability is inconsistent^8,18,19^. The present study found significant differences in the intra-individual pre-post comparisons for the condition SM (Fig. 3). In this within-subject comparison, the RRI, RMSSD, and LF/HF parameters indicate an increased parasympathetic withdrawal for SM in the post-exercise period compared to the initial pre-exercise phase. In contrast, there are no changes in the respective parameters for LP. DFAa1 did not show any differences between individuals when comparing intra-individually pre- and post-exercise values in the SM and LP conditions. Consequently, the lower RMSSD values observed in the SM condition can be attributed to a greater withdrawal of parasympathetic activity and altered sympathetic-parasympathetic balance, indicated by the LF/HF ratio, post-exercise. Nevertheless, in the pre-post comparison (Table 4), the RMSSD did not differ between the conditions. Thus, the standing body position leads to significantly lower parasympathetic activity during the pre-exercise period, which remains unchanged in the post-exercise phase compared to the sitting position. The authors of this paper suggest that orthostasis has a significant influence on the vagal differences observed in the respective pre-exercise period (Table 3) and in post-exercise regulation (Table 2). In LP, RMSSD (Table 2), therefore, increases significantly more in the post-exercise period compared to SM. Rezek et al. (2006) postulate that an increase in sympathetic activity following the end of strength training stabilizes blood pressure, with decreased stroke volume and only a slight increase in TPR. Consistently, the lower expression of RRI in SM is the result of lower parasympathetic influence and/or possibly a higher sympathetic activity in the pre-exercise phase (Table 3). However, the recovery status of autonomic activity returns to pre-exercise level within five minutes after LP. According to the current evidence, only SM demonstrates increased parasympathetic withdrawal in the intra-individual pre-post comparisons^8,18,19^. This intra-individual post-exercise regulation cannot be observed for LP^39^.

In summary, the LP condition showed that there appears to be no intra-individual autonomic change after strength exercise compared to pre-exercise^39^. In contrast, the comparison reveals a significant parasympathetic withdrawal following strength exercise in SM^8,18,19^, which is likely associated with the standing position and higher muscular demands at rest and during the exercise. At comparative intensities (50% 3-RM), strength exercise in the seated position within LP showed a higher level of parasympathetic return.

### Derivation for health sports

Standing in a squatting position increases the demands on the cardiopulmonary and vascular systems, leading to a significantly greater withdrawal of parasympathetic activity compared to seated exercise before and immediately after the workout. This finding is crucial for patients with coronary artery disease, valvular defects, arterial vascular disease, and/or cardiac arrhythmias, as it suggests a way to reduce risk while performing leg strength training exercises. The choice between these two types of exercise—SM and LP—should be made individually, taking into account the patient’s risk profile and specific needs.

### Study limitations

Several limitations should be acknowledged, as they may influence the interpretation of the study’s findings. First, the sample size was relatively small and focused exclusively on active female participants to minimize potential differences in cardiopulmonary function and muscle performance due to sex. Consequently, the findings may not be easily interpreted or generalized to older or diseased populations. Therefore, it is essential to conduct future studies with a more diverse range of participants. It is important to note that the evaluation of the 5-minute intervals includes both the 1-minute afterload phase and the 25-second phase for assuming the starter position (not shown in Fig. 1). Nonetheless, this trial represents the most extensive randomized crossover study investigating acute autonomic responses during matched standing and sitting strength training exercises in women. The different body positions during the resting periods, such as sitting and standing, may have influenced both the exercise and the post-exercise results. Future research should clarify this influence.

## Conclusions

The present results confirm that, in addition to the metabolic and neuro-muscular requirements of training, the body position also has an important influence on autonomic regulation. A seated body position promotes the parasympathetic influence, in contrast to the orthostatic influence of a standing position in the context of leg strength training, accompanied by higher correlation properties and less complexity in heart rate time series. Reduced parasympathetic activity during standing is a plausible mechanism for compensatory responses to cardiovascular afterload regulation. Since sex-specific differences in blood pressure regulation have already been shown^40^studies should also compare female and male populations. The results should also be examined in the context of various diseases.

## Data Availability

The datasets used and/or analysed during the current study are available from the corresponding author on reasonable request.

## Abbreviations

DFAa1: Alpha1 of detrend fluctuation analysis
ECG: Electrocardiogram
HR: Heart rate
HRV: Heart rate variability
LF/HF: Ratio of low frequency and high frequencies
LP: Leg press
SM: Smith machine
3-RM: 3-repetition maximum test
RMSSD: Root mean square of successive differences
RRI: RR intervals
